# F230. COMPARISON OF PALIPERIDONE PALMITATE 3-MONTH AND PALIPERIDONE PALMITATE 1-MONTH FORMULATION FOR NEGATIVE SYMPTOMS IN SCHIZOPHRENIA: A PHASE 3 NON-INFERIORITY STUDY

**DOI:** 10.1093/schbul/sby017.761

**Published:** 2018-04-01

**Authors:** Maju Mathews, Srihari Gopal, Arun Singh, Jagadish Gogate, Edward Kim, Katalin Pungor

**Affiliations:** 1 Janssen Research & Development, LLC; 2 Janssen Scientific Affairs, LLC

## Abstract

**Background:**

Negative symptoms of schizophrenia are key predictors of long-term disability. It is important to understand whether treatment with long-acting injectable antipsychotics can improve negative symptom psychopathology. Paliperidone palmitate 3-month formulation (PP3M) provides a sustained release of paliperidone, permitting a significantly extended dosing interval of only 4 doses per year in patients with schizophrenia. The efficacy of PP3M as assessed by relapse rate is comparable to the paliperidone palmitate 1-month formulation (PP1M). The purpose of this post-hoc analysis was to compare the improvement in negative symptoms in patients treated with PP1M and PP3M.

**Methods:**

Data from a randomized, double-blind (DB), parallel-group, multicenter, phase 3 study in patients with schizophrenia were analyzed. Patients aged 18 to 70 years with schizophrenia (DSM-IV-TR) and a total Positive and Negative Syndrome Scale (PANSS) score of 70–120 at screening were enrolled. After screening (3 weeks), patients entered a 17-week open-label (OL) phase, to receive PP1M (day 1 [150 mg eq. deltoid], day 8 [100 mg eq. deltoid], weeks 5, 9 and 13 [50, 75, 100, or 150 mg eq., deltoid/gluteal]) and entered a 48-week DB phase and were randomized (1:1) to receive fixed doses of either PP1M (50, 75, 100, or 150 mg eq., stabilized in OL) or PP3M (175, 263, 350, or 525 mg eq.) in deltoid or gluteal muscle until they relapsed or withdrew from study. The PANSS total scores with emphasis on 7-item negative subscale scores for PP1M vs PP3M were assessed.

**Results:**

Of 1429 patients enrolled, 1016 were randomized to receive PP3M (n=504) or PP1M (n=512) in DB phase. Majority of patients were men and white (both 55%), with a mean (SD) age of 38.4 (11.86) years. At baseline, the mean (SE) negative subscale total was 23.2 (0.12), indicating a moderate to severe level of negative symptoms. Negative subscale and negative symptoms factor scores showed continuous improvements throughout the OL and double-blind phases of the study - mean (SD) at OL baseline and DB endpoint for total negative subscale score and symptom factor score were 23.2 (4.60) and 22.3 (4.87), and 15.9 (4.99) and 14.9 (4.81), both R2:0.16, respectively. The mean (SD) PANSS negative subscale score changes from DB baseline for PP1M vs PP3M were similar over time (mean change from baseline to DB endpoint was -1.4 (3.67), R2:0.06 vs -1.4 (3.63), R2:0.05).

**Discussion:**

Development of an LAI antipsychotic with less frequent dosing than those currently available would be of potential advantage to patients, caregivers, and prescribers. PP3M and PP1M demonstrated consistent and similar efficacy in patients with moderate to severe negative symptoms of schizophrenia over the observed timepoints, including impact on patients with predominantly negative symptoms. Longer continuous treatment with PP3M showed greater benefit. This indicates that long-acting therapies are associated with continued improvement in negative symptoms over time. Treatment with LAIs for longer than a year was associated with the greatest improvements in negative symptoms.

